# Incidence and Predictors of Pregnancy among Women on ART in Debre Markos Referral Hospital, Northwest Ethiopia:* A Five-Year Retrospective Cohort Study*

**DOI:** 10.1155/2017/3261205

**Published:** 2017-06-05

**Authors:** Maru Meseret, Alemayehu Shimeka, Alemayehu Bekele

**Affiliations:** ^1^Amhara National Regional State Health Bureau, East Gojjam Zonal Health Department, Bahir-Dar, Ethiopia; ^2^Department of Epidemiology and Biostatistics, College of Medicine and Health Sciences, University of Gondar, Gondar, Ethiopia; ^3^Ethiopian Public Health Association, Addis Ababa, Ethiopia

## Abstract

Globally, death of women due to HIV/AIDS related causes during pregnancy or within 42 days after pregnancy was estimated to be 37,000. In Ethiopia, 42,900 pregnant women living with HIV gave birth in the year 2011. This study was aimed to assess incidence and predictors of pregnancy among women on ART in Debre Markos Referral Hospital, Northwest Ethiopia. A retrospective cohort study was conducted using data recorded from September 2011 to August 2015. Data was extracted from February to March, 2016, from 1,239 records and analyzed using SPSS version 16. A Kaplan-Meier survival analysis was used to estimate the probabilities of being pregnant. The Cox proportional hazards model was done and results were expressed using hazard ratios with 95% CI. A total of 1,239 women on ART were included in the study. The incidence of pregnancy was 49.2 per 1,000 person-years. Living in rural, being married, being widowed, being unemployed, and having <2 children at enrollment were found to be positively associated with being pregnant. The incidence of pregnancy among women on ART was found to be considerable. Provision of family planning and other reproductive health interventions have to be coupled with the ART service to address the problem.

## 1. Introduction

An estimated 35.3 million people were living with human immune virus (HIV) worldwide, from which 3.3 million are children. Approximately, 57% of all adults living with HIV/AIDS were women. The situation is very serious in Sub-Saharan African countries where 75% of all people with HIV in the globe are living [1]. According to UNAIDS 2013 global report, women of reproductive age in Sub-Saharan Africa were disproportionately affected by HIV/AIDS; 80% of the nearly 13 million women living with HIV worldwide live in this region [2]. According to the 2011 Ethiopian Demographic and Health Survey (EDHS), the prevalence of HIV among women aged 15–49 years was 1.9% and the prevalence increases with increasing women's age [3]. In low and middle income countries, an estimated 1.5 million women living with HIV were pregnant. Globally, death of women due to HIV/AIDS related causes during pregnancy or within 42 days after pregnancy ends was estimated to be 37,000 [4].

Women who are living with HIV should access treatment based on WHO treatment guidelines. Nowadays, antiretroviral therapy (ART) is widely available in areas with meager resources. Providing ART for women living with HIV gives women good physical functioning and an opportunity to dream a better future. ART improves health of mothers and these mothers may be motivated to have more children [5]. In most circumstances, even though it is their right to have children, women are discouraged from getting pregnant while on ART due to the fact that giving birth from HIV positive mother could result in HIV positive babies. If proper care for the mothers is not in place, maternal death due to complications related to HIV and pregnancy can be very high [6].

A meta-analysis of 23 studies showed that HIV positive women have eight times risk of pregnancy related death compared to women without HIV. One in four pregnancy related deaths in Sub-Saharan Africa was attributable to HIV [7]. Despite the fact that providing treatment to women living with HIV/AIDS has a tremendous effect on the health of a mother and her newborn, a birth from HIV positive mother who is on ART may end up with not only HIV positive baby but also with maternal death. Studies have shown that, even in the presence of ART, maternal mortality rates have been reported to be five times higher in HIV infected women than in uninfected women and HIV/AIDS was responsible for at least 20% of all deaths, a figure that is higher than any direct obstetric cause [8].

A study in Uganda estimates that, even with a projected scale-up of antiretroviral therapy (ART) based prevention of mother to child transmission (PMTCT), unwanted pregnancies among women with HIV account for almost a quarter of all HIV positive infants and about a fifth of pediatric AIDS deaths [9]. HIV positive women who seek induced abortion may be at greater risk of morbidity than HIV uninfected women [10]. ART by itself may have a negative effect on birth outcomes such as prematurity or low birth weight [11].

Women living with HIV desire additional pregnancies and thus should be assisted to conceive, carry a pregnancy to term, deliver, and care for the resulting child. However, in other cases, women living with HIV become pregnant despite not desiring. Globally, many women lack access to family planning information and modern contraceptive methods; as a result, many pregnancies are unintended. In countries with the highest HIV burden, unintended pregnancies account for 14–58% of all births, and rates of unintended pregnancy among women living with HIV are high [12].

Evidences from Canada and Malawi indicated that the desire to have more children among mothers living with HIV was 69% and 17%, respectively [13, 14]. The magnitude of the desire to have more children in Ethiopia varies from place to place. The desire to have more children among mothers living with HIV in North Wollo, Addis Ababa, Harari region, and Oromia region was 15.7%, 44%, 53%, and 92%, respectively [15–18]. The tendency of getting pregnant and having HIV negative baby is highly improved due to the introduction of ART service which reduces mother to child transmission of HIV resulting in increased incidence of pregnancy among HIV positive women [6, 19–23].

However, because of the desire to have more children or unmet needs of family planning services, the incidence of pregnancy among women living with HIV in Ethiopia was likely to be high. Therefore, the main aim of this study was to assess the magnitude of incidence of pregnancy and predictors of it among women on HIV treatment in Debre Markos Referral Hospital.

## 2. Rational of the Study

Introduction of antiretroviral therapy to women living with HIV highly improves lifestyle and the desire to have children. However, evidences have shown that even in the presence of ART maternal mortality rates have been reported to be five times higher in HIV infected women compared to uninfected women. In Ethiopia being one of the developing countries, pregnancy related complications among women on ART are among the many health challenges. Even though the country is aggressively constructing and equipping health facilities with staff and equipment, still morbidity and mortality due to complications related to pregnancy among pregnant women on ART are very high. As far as the investigators search, there is scarcity of evidences on incidence of pregnancy among reproductive age group mothers taking ART in hospitals of Ethiopia. Considering resource limitations, this study was conducted in one of the hospitals which is Debre Markos Referral Hospital.

## 3. Methods and Materials

### 3.1. Study Design

Institutional based retrospective cohort study design using five-year data from September 2011 to August 2015 was employed.

### 3.2. Study Area and Period

This study was conducted in Debre Markos Referral Hospital. Debre Markos is a city located 300 kilometers far from Addis Ababa, the capital of Ethiopia, and 256 km from Bahir-Dar, a capital of Amhara National Regional State. The hospital provides health service to more than 3.5 million populations. Currently, about 100 health centers and two district hospitals are available in the catchment area of the referral hospital. There are 109 nurses, 3 health officers, 16 general practitioners, and one emergency surgeon and five specialists. Gynecologic and obstetric ward has 19 midwives, one gynecologist, and one emergency surgeon. About 8,136 patients are admitted per year, 34% of them in obstetric ward [24]. A database from I-TECH prepared excel sheet has been seen and about 1,239 women were registered on ART from September 2011 to August 2015.

#### 3.2.1. Population

All women on ART in Debre Markos Referral Hospital catchment area were the source population for this study. All women who were on antiretroviral treatment and registered for follow-up care from September 2011 to August 2015 in the hospital were taken as the study population for this study.

#### 3.2.2. Inclusion and Exclusion Criteria

Mothers who were getting antiretroviral treatment in the hospital from September 2011 to August 2015 were included in the study. However, mothers who were getting pre-ART care and started ART based on option B+ and mothers with more than one pregnancy during the follow-up time in the hospital in the specified period were excluded from the study.

#### 3.2.3. Variables

The dependent variable of the study was incidence of pregnancy which was labeled as yes and/or no. Independent variables included sociodemographic characteristics such as age, residence, marital status, occupation, educational status, number of children, and clinical characteristics like CD4 count, WHO staging, weight, BMI, and functional status of women on ART.

### 3.3. Operational Definitions

#### 3.3.1. Incidence of Pregnancy

In this research, incidence of pregnancy was considered as the first pregnancy after ART enrollment within the follow-up time.

#### 3.3.2. Time to Event

In this research, time to event was defined as the time from enrollment on ART to the conception of the first pregnancy.

#### 3.3.3. Exposed

According to this research, married women were considered as exposed to pregnancy while on ART.

#### 3.3.4. Unexposed

Unmarried women were considered as unexposed to pregnancy while on ART.

#### 3.3.5. Sampling Technique

The total records in the hospital among women of reproductive age group were 1,239. All the records of mothers on ART follow-up during the five consecutive years with complete information were used to determine incidence and predictors of pregnancy among women on ART.

#### 3.3.6. Data Collection Tools

A structured data collection checklist was prepared in English to extract data from the records.

#### 3.3.7. Data Collection Procedures

Data was extracted from main excel record of the hospital. Data elements which were not included in the excel sheet were collected from ART intake forms and cards. The data was collected by two health informatics professionals of the hospital working on ART database and supervised by one B.S. nurse.

#### 3.3.8. Data Quality Control

Training was given for the data collectors and the supervisor before the actual data collection. Completeness of data was checked in each day of activity and the necessary feedback was offered to data collectors the next morning. Besides this, the principal investigators and an experienced data clerk carefully entered and thoroughly cleaned the data before the commencement of the analysis.

### 3.4. Data Management and Analysis

Date extracted from the data set was entered into excel sheet. Completeness of the excel sheet data set was checked thoroughly and exported to SPSS version 16 for analysis. Descriptive statistics and incidence rate of pregnancy in person-years of follow-up were reported. A Kaplan-Meier curve with hazard and survival functions was used to estimate the probabilities of becoming pregnant. The Cox proportional hazards model was used and results were expressed as hazard ratios with 95% CIs. Variables with *p* value ≤ 0.2 at Kaplan-Meier survival analysis were entered into multivariable Cox proportional hazards model to identify the independent predictors of pregnancy. Adjusted hazard ratio with 95% CI was used to report the findings.

### 3.5. Ethical Considerations

A letter of ethical clearance was obtained from Ethiopian Public Health Association and Research Ethics Committee. Informed consent was obtained from East Gojjam Zonal Health Department and Debre Markos Referral Hospital. All data sets were extracted from ART database and stored with password. Full information was kept confidential.

## 4. Results

### 4.1. Sociodemographic Characteristics of Women on ART

A total of 1,951 records were reviewed. Out of these, 681 and 31 records had missing data on date of enrollment to ART and the last date of menstrual period, respectively, and thus were excluded from the analysis. Therefore, a total of 1,239 records had complete data and were considered for the final analysis. The average period for follow-up was 2.23 years with SD of 1.54. The mean age with SD of study participants was 30 ± 10.85 years. Among women included in the study, 550 (44.4%) were aged between 25 and 34 years, 957 (77.2%) were urban dwellers, 489 (38.7%) were married, 590 (47.6%) were unable to read and write, 692 (55.9%) have less than two children, and 975 (78.7%) were unemployed (Table 1).

### 4.2. Clinical Characteristics of Women on ART

Among the study participants, 562 (45.4%) had CD4 count less than 200 cells/mm^2^ at enrollment, 643 (51.9%) had weight less than 50 kilograms, 440 (35.5%) were at 3rd clinical stage, 908 (73.3%) had a BMI of 18.5 to 25, and 1,030 (84.2%) of them had a functional status of working at ART enrollment (Table 2).

### 4.3. Incidence of Pregnancy

Initially, a total of 1,239 women on ART were included in the study from September 2011 to August 2015 at different entry to the cohorts. The highest number of enrollment to ART was registered during 2011 which was 355 (28.7%). The overall incidence of pregnancy was found to be 49.2 per 1000 person-years (136 pregnancies in a total of 2762.55 years of observation). The incidence of pregnancy among exposed women was 74.6 per 1000 person-years (79 pregnancies in a total of 1058.88 years of observation) while it was 33.46 per 1000 person-years (57 pregnancies in a total of 1703.67 years of observation) in unexposed. The highest rate of pregnancy was registered among the cohorts of 2015 with 109.5 per 1000 person-years.

### 4.4. Kaplan-Meier Survival Analysis

**Figure 1:**
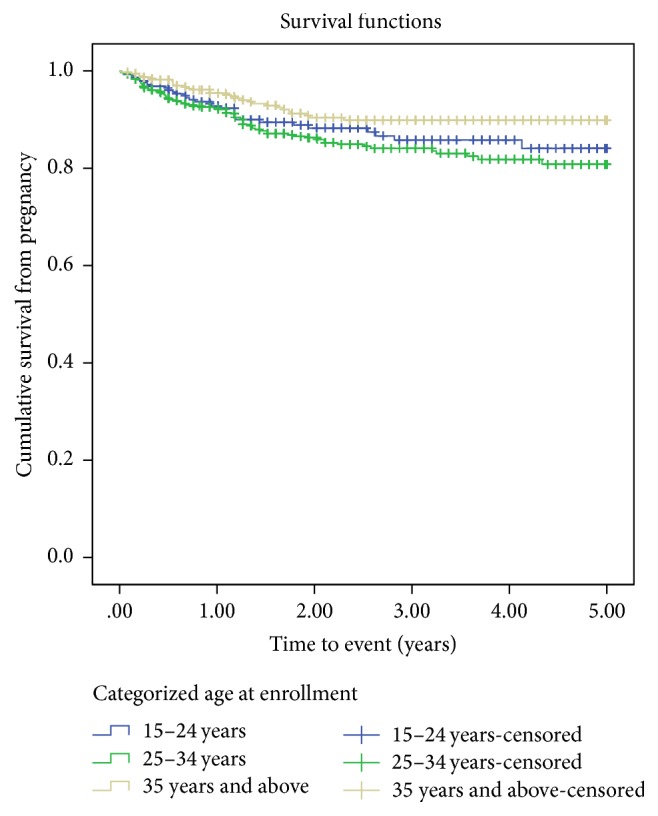
Kaplan-Meier curves of pregnancy comparing age among women receiving ART treatment from Sep. 2011 to Aug. 2015 in Debre Markos Referral Hospital, Northwest Ethiopia, 2016 (*N* = 1,239).

**Figure 2:**
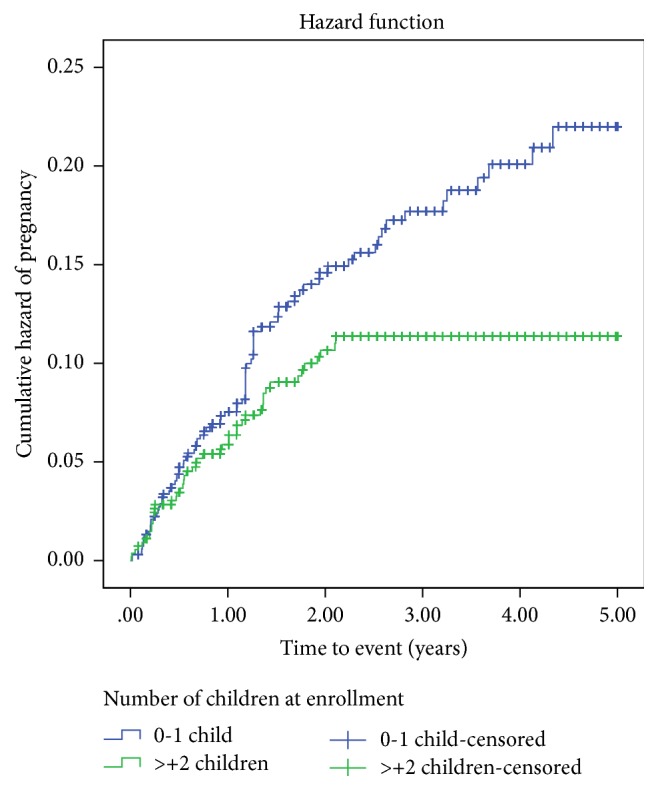
Kaplan-Meier curves of pregnancy showing hazard of pregnancy among women receiving ART treatment from Sep. 2011 to Aug. 2015 in Debre Markos Referral Hospital, Northwest Ethiopia, 2016 (*N* = 1,239).

The cumulative probability of surviving from pregnancy was higher among women aged 35 and above with mean survival time 4.60 years, 95% CI (4.47–4.74). This means the cumulative hazard of pregnancy was higher among women aged 25 to 34 years (Figure 1). At Kaplan-Meier analysis, statistically significant association was observed with incidence of pregnancy at *p* value < 0.05. At the beginning of the study, the probability of getting pregnant for all women was almost the same. However, the risk of getting pregnant among women having less than two children was increasing with follow-up time. The cumulative probability of surviving from pregnancy for all women was almost the same, but after follow-up it was higher among women having two and above children than those having less than two children with mean survival time of 4.56 years, 95% CI (4.44–4.68) and 4.33, 95% CI (4.20–4.46), respectively. At Kaplan-Meier analysis, statistically significant association was observed with incidence of pregnancy at *p* value < 0.05 (Figure 2).

With respect to residence, the cumulative hazard of pregnancy at year one for urban and rural residents was 5% and 9%, respectively, whereas the cumulative probability of surviving at year one for urban and rural residents was 89% and 85% with mean survival time 4.48 years, 95% CI [4.38–4.57], and 4.28 years, 95% CI [4.08–4.49], respectively. The cumulative hazard of pregnancy at year one for women with marital status never married, married, widowed, and divorced was 4%, 9%, 6%, and 3%, respectively, whereas the cumulative probability of surviving at year one for women with marital status never married, married, widowed, and divorced was 94%, 82%, 91%, and 90%, respectively. At Kaplan-Meier analysis, statistically significant association was observed with incidence of pregnancy at *p* value < 0.05. The lowest cumulative probability of surviving from pregnancy had been observed among women with marital status married in the remaining years. Throughout follow-up period, the risk of getting pregnant was increasing among women who were unable to read and write compared to those with primary and above education. At Kaplan-Meier analysis, statistically significant association was observed with incidence of pregnancy at *p* value < 0.05. Cumulative probability of getting pregnant among unemployed mothers was higher during early phase of the study and continued to increase throughout follow-up years compared to employed mothers whereas the cumulative probability of surviving from pregnancy among employed mothers is higher than unemployed with mean survival time of 4.63 years, 95% CI (4.46–4.78), and 4.38 years, 95% CI (4.28–4.48), respectively. At Kaplan-Meier estimate, statistically significant association was observed with incidence of pregnancy at *p* value < 0.05.

### 4.5. Predictors of Pregnancy among Mothers on ART

Variables with a *p* value of <0.2 at Kaplan-Meier estimate were used to assess the actual effect on incidence of pregnancy. Backward stepwise method was used. The risk of getting pregnant among rural residents women was 17.87 times higher compared to urban counterparts [AHR = 17.87, 95% CI (10.72–29.79)]. Married, widowed, and divorced women had 5.35, 3.65, and 4.34 times higher risk of pregnancy compared to women who had never married [AHR = 5.35, 95% CI (2.14–13.36), AHR = 3.65, 95% CI (1.06–12.49), and AHR = 4.34, 95% CI (1.42–13.22), resp.]. Women who had less than two children had 2.05 times higher risk of pregnancy compared to those who had two or more children [AHR = 2.05, 95% CI (1.31–3.20)]. Unemployed women had 4.37 times higher incidence of pregnancy compared to employed counterparts [AHR = 4.37, 95% CI (1.91–9.98)] (Table 3).

## 5. Discussion

This study has aimed at assessing the incidence of pregnancy and its predictors. Accordingly, the overall incidence of pregnancy was found to be 49.2 per 1000 person-years. This finding was lower than the findings of other studies such as United States of America [6], Sub-Saharan Africa [25], Uganda, and Ethiopia [26–30] with 74, 78, 90.7, and 65 per 1000 person-years, respectively.

The possible reasons for the explanation might be due to differences in the access and quality of the services, the size of records reviewed, the study period, and differences in inclusion and exclusion criteria. The risk of getting pregnant was 17.87 times higher among the rural women compared to their urban counterparts. This could be due to the fact that, in rural areas, the accessibility of family planning services was lower in the community and for mothers on ART in particular.

Married, widowed, and divorced women were 5.35, 3.65, and 4.34 times higher compared to women who never married, respectively. This finding was in line with the findings from United States of America and Sub-Saharan Arica [6, 25]; however, it was not consistent with the findings of a study of Western Uganda [26]. The possible explanation for the discrepancies might be due to differences in the size of records reviewed, the study period, and differences in inclusion and exclusion criteria.

The risk of pregnancy was 2.05 times higher among mothers who had zero or one child compared to those who had two and above children. This finding was in line with the findings of a study done in Western Uganda and Ethiopia [26, 30]. Women who had less than two children might have high fertility desire compared to those having two or more children. This might be due to the fact that mothers who had two or more children had better utilization of family planning services. The risk of pregnancy among unemployed women was also 4.37 times higher compared to the employed. It was in line with the findings of a study done in Western Uganda [26]. The possible justification of this finding might be due to the fact that in most circumstances in Ethiopia employed women are those with better educational status; in turn those women having better education may have a chance to access information from different types of media.

## 6. Limitations of the Study

Since this research was done using secondary data, all variables such as disclosure to sexual partner, adherence to ART, family planning methods uptake, open discussion with sexual partner, and other reproductive health issues which can possibly affect pregnancy among women on ART were not included. In addition to this, excluding charts with incomplete records could have an effect on the validity of the findings.

## 7. Conclusion

In light of the findings of this study, the authors forwarded the following remarks. The incidence of pregnancy among women on ART in Debre Markos Referral Hospital was found to be considerable. Living in rural areas, being married, widowed, and divorced, being unemployed, and having less than two children at enrollment on ART were found to be predictors of incidence of pregnancy among women on ART.

## 8. Recommendation

Considering the findings of this study, the Regional Health Bureau, Zonal Health Department, and health institutions should ensure that reproductive health services have to be well integrated into the HIV care system. Effective counseling strategies have to be designed focusing on rural, married, widowed, and divorced, unemployed mothers and mothers with less than two children at enrollment to reduce the risks of getting pregnant.

## Figures and Tables

**Table 1:** Sociodemographic characteristics of women receiving ART treatment from Sep. 2011 to Aug. 2015 in Debre Markos Referral Hospital, Northwest Ethiopia, 2016 (*N* = 1239).

Variables	Pregnancy status		Total frequency
	No	Yes	
Age			
15–24 years	267 (89.3%)	32 (10.7%)	299 (24.1%)
25–34 years	476 (86.5%)	74 (13.5%)	550 (44.4%)
≥35 years	360 (92.3%)	30 (7.7%)	390 (31.5%)
Place of residence			
Urban	861 (90%)	96 (10%)	957 (77.2%)
Rural	242 (85.8%)	40 (14.2%)	282 (22.8%)
Marital status			
Never married	239 (95.2%)	12 (4.8%)	251 (20.3%)
Married	400 (83.5%)	79 (16.5%)	479 (38.7%)
Widowed	258 (91.5%)	24 (8.5%)	282 (22.8%)
Divorced	206 (90.7%)	21 (9.3%)	227 (18.3%)
Educational status			
Unable to read and write	493 (83.6%)	97 (16.4%)	590 (47.6%)
Primary (1–8)	224 (94.5%)	13 (5.5%)	237 (19.1%)
Secondary (9–12)	197 (92.1%)	17 (7.9%)	214 (17.3%)
Certificate and above	189 (95.5%)	9 (4.5%)	198 (16%)
Occupational status			
Unemployed	858 (88%)	117 (12%)	975 (78.7%)
Employed	245 (92.8%)	19 (7.2%)	264 (21.3%)
Number of children at enrollment			
0-1 child	604 (87.3%)	88 (12.7%)	692 (55.9%)
≥2 children	499 (91.2%)	48 (8.8%)	547 (44.1%)

**Table 2:** Clinical characteristics of women receiving ART treatment in Debre Markos Referral Hospital, Northwest Ethiopia, Sep. 2011 to Aug. 2015 (*N* = 1239).

Variables	Pregnancy status	
	No	Yes
CD4 count at enrollment		
<200 cell/mm^2^	488 (86.8%)	74 (13.2%)
200–350 cell/mm^2^	446 (91%)	44 (9%)
>350 cell/mm^2^	169 (90.4%)	18 (9.6%)
Functional status at enrollment		
Working	913 (88.6%)	117 (11.4%)
Ambulatory	163 (91.6%)	15 (8.4%)
Bedridden	15 (100%)	0 (0%)
Weight at enrollment		
≤50 kg	580 (90.2%)	63 (9.8%)
51–60 kg	307 (89%)	38 (11%)
61–70 kg	100 (84%)	19 (16%)
≥71 kg	116 (87.9%)	16 (12.1%)
WHO clinical stage at enrollment		
Clinical stage 1	367 (89.5%)	43 (10.5%)
Clinical stage 2	258 (85.7%)	43 (14.3%)
Clinical stage 3	395 (89.8%)	45 (10.2%)
Clinical stage 4	83 (94.3%)	5 (5.7%)
BMI at enrollment		
<18.5	127 (91.4%)	12 (8.6%)
18.5–25	802 (88.3%)	106 (11.7%)
>25	174 (90.6%)	18 (9.4%)

**Table 3:** Cox's proportional hazards models examining predictors of incidence of pregnancy with 95% CIs, among women on ART in Debre Markos Referral Hospital, Northwest Ethiopia, Sept. 2011 to Aug. 2015 (*N* = 1,239).

Variable	Status of pregnancy		CHR (95% CI)	AHR (95% CI)
	No	Yes		
Place of residence				
Urban	861 (90%)	96 (10%)	1	1
Rural	242 (85.8%)	40 (14.2%)	1.40 (0.97–2.02)	17.87 (10.72–29.79)
Age				
15–24	267 (89.3%)	32 (10.7%)	1	
25–34	476 (86.5%)	74 (13.5%)	1.22 (0.81–1.85)	
≥35 years	360 (92.3%)	30 (7.7%)	0.68 (0.41–1.12)	
Marital status				
Never married	239 (95.2%)	12 (4.8%)	1	1
Married	400 (83.5%)	79 (16.5%)	3.61 (1.97–6.64)	5.35 (2.14–13.36)
Widowed	258 (91.5%)	24 (8.5%)	1.81 (0.90–3.62)	3.65 (1.06–12.49)
Divorced	206 (90.7%)	21 (9.3%)	2.13 (1.05–4.34)	4.34 (1.42–13.22)
Educational status				
Unable to read & write	493 (83.6%)	97 (16.4%)	3.83 (1.93–7.59)	2.28 (0.89–5.84)
Primary (1–8)	224 (94.5%)	13 (5.5%)	1.24 (0.53–2.90)	0.77 (0.24–2.48)
Secondary (9–12)	197 (92.1%)	17 (7.9%)	1.81 (0.80–4.06)	1.31 (0.37–4.61)
Certificate and above	189 (95.5%)	9 (4.5%)	1	1
Occupational status				
Unemployed	858 (88%)	117 (12%)	1.72 (1.06–2.80)	4.37 (1.91–9.98)
Employed	245 (92.8%)	19 (7.2%)	1	1
Number of children at enrollment				
0-1 child	604 (87.3%)	88 (12.7%)	1.55 (1.09–2.21)	2.05 (1.31–3.20)
≥2 children	499 (91.2%)	48 (8.8%)	1	1

Note: level of significance was considered at a cutoff point of the *p* value of 0.05.
